# Patients Follow-up for Spinal Epidural Abscess as a Critical Treatment Plan Consideration

**DOI:** 10.7759/cureus.35058

**Published:** 2023-02-16

**Authors:** Rhett MacNeille, Johnson Lay, Jacob Razzouk, Shelly Bogue, Gideon Harianja, Evelyn Ouro-Rodrigues, Caleb Ting, Omar Ramos, Jennifer Veltman, Olumide Danisa

**Affiliations:** 1 Department of Orthopaedic Surgery, Loma Linda University Medical Center, Loma Linda, USA; 2 Department of Orthopaedics, School of Medicine, California University of Science and Medicine, Colton, USA; 3 Department of Orthopaedic Surgery, Loma Linda University School of Medicine, Loma Linda, USA; 4 Department of Orthopaedics, School of Medicine, University of California Riverside School of Medicine, Riverside, USA; 5 Department of Infectious Diseases, Loma Linda University Medical Center, Loma Linda, USA

**Keywords:** post-operative care, surgery spine, patient follow-up, infectious disease control, treatment of spinal epidural abscess

## Abstract

Introduction: Spinal epidural abscess (SEA) is a rare process with significant risk for morbidity and mortality. Treatment includes an extended course of antibiotics with or without surgery depending on the clinical presentation. Both non-operative and surgically treated patients require close follow-up to ensure the resolution of the infection without recurrence and/or progression of neurologic deficits. No previous study has looked specifically at follow-up in the SEA population, but the review of the literature does show evidence of varying degrees of difficulty with follow-up for this patient population.

Methods: This retrospective review looked at follow-up for 147 patients with SEA at a single institution from 2012 to 2021. Statistical analyses were performed to assess differences between groups of surgical versus non-surgical patients and those with adequate versus inadequate follow-up.

Results: Sixty-two of 147 (42.2%) patients had inadequate follow-up (less than 90 days) with their surgical team, and 112 of 147 (76.2%) patients had inadequate follow-up (less than 90 days) with infectious disease (ID). The primary statistically significant difference between patients with adequate versus inadequate follow-up was found to be surgical status with those treated surgically more likely to have adequate follow-up than those treated non-operatively.

Conclusion: Improved follow-up in surgical patients should be considered as a factor when deciding on surgical versus non-operative treatment in the SEA patient population. Extra efforts coordinating follow-up care should be made for SEA patients.

## Introduction

Spinal epidural abscess (SEA) is a rare, pyogenic infection involving the space between the dura and spinal periosteum with an incidence between 0.2 and 2 cases per 10,000 admissions, trending higher in recent decades [1]. The four phases of the clinical presentation have been described as fever and back pain, then nerve root pain, weakness, and finally paralysis [1]. Treatment involves a long course of antibiotics with or without surgical intervention [2-6].

Outcomes and treatment success rates reported in the literature paint a grim picture. Heusner’s series reported a 45% rate of either death or residual paralysis [1]. Non-operative failure has been reported as ranging from 27% to 41% [7]. Risk factors associated with higher rates of non-operative treatment failure include leukocytosis greater than 12.5, C-reactive protein (CRP) greater than 115 mg/L, diabetes mellitus (DM), positive blood cultures, age greater than 65 years old, methicillin-resistant *Staphylococcus aureus* (MRSA), active malignancy, and pathologic/compression fracture at the effected levels [7]. Shah et al. also noted the dorsal location of an abscess to be somewhat more protective from failure than a ventral abscess [7]. Recurrence rates have been reported to range from 6% to 30% [8].

With these humbling statistics, outpatient follow-up has been highlighted as a vital part of the treatment course for this patient population, ensuring the resolution of the infection while monitoring closely for complications, treatment failure, and/or recurrence. The purpose of this study is to evaluate the follow-up rates of patients with SEAs, the potential factors associated with follow-up failure, and to consider the potential implications these findings may have on formulating treatment plans. To the authors’ knowledge, this is the first publication to specifically address follow-up in the SEA patient population.

## Materials and methods

This was designed as a single institution retrospective study, approved by the Institutional Review Board (#5200262). An initial chart search was performed from years 2012 to 2021 using the International Classification of Diseases (ICD) 9 and ICD 10 codes for SEA as well osteomyelitis and discitis. Inclusion criteria included any patient with a SEA that was able to be verified with an available magnetic resonance imaging (MRI). Exclusion criteria included lack of MRI records or epidural abscess related to post-operative infection. A total of 147 patients were identified from the dataset as meeting the criteria for this study. Follow-up rates with both the surgical team (orthopedic surgery or neurological surgery) and infectious disease (ID) specialists were collected. Patients were classified as operatively versus non-operatively treated based on whether or not the patient received operative intervention for their pathology. We also collected associated demographic and clinical data.

Four different statistical analyses were performed to look for potential differences between groups. First, differences between surgically treated patients versus non-operatively treated patients were compared. Our second and third comparisons looked at differences between patients with less than 90 days of follow-up versus 90 or more days of follow-up with their surgical team and their ID team respectively. Ninety days was selected as the cutoff point because it was considered by the authors to be the minimum necessary time to achieve what could reasonably be considered an ‘adequate’ follow-up. Our fourth and final analysis compared surgically treated versus non-operatively treated patients and their rates of expected follow-up. Expected follow-up was defined as surgeon follow-up for surgically treated patients and ID follow-up for non-operatively treated patients. While all epidural abscess patients should ideally have follow-ups with both the surgery team and ID specialists, this expected follow-up definition was felt to be the minimum reasonable expectation for patients and the most fair and conservative comparison.

Data collection and visualization were performed using Microsoft Excel version 16.58 (Microsoft Corporation, 2022, Redmond, WA, USA). Statistical Product and Service Solutions (SPSS) version 28 (IBM Corporation, 2021, Armonk, NY, USA) was utilized for all subsequent statistical analyses. Statistical significance was defined as P < 0.05. Correlation coefficients were defined using the commonly categorized values of weak, moderate, and strong corresponding to value ranges of 0-0.3, 0.3-0.7, and 0.7-1, respectively. Descriptive statistics utilized means, standard deviations (SD), 95% confidence intervals (CI), mean differences, and percentages for demographic and anthropometric data. Independent sample t-tests with Levene’s test for equality of variances were used to identify differences in numerical variables. Pearson’s Chi-squared tests with point-biserial correlations (Phi and Cramer’s V), relative risk (RR), and odds ratio (OR) were used to identify differences for categorical variables.

## Results

Surgically treated versus non-operatively treated patients

Of our 147 patients, 103 were treated surgically while 44 had non-operative treatment. Surgically treated patients were significantly more likely to have ≥90 days of surgical follow-up than non-operatively treated patients (p-value <0.001). This did not hold true for ID follow-up. There were no other significant differences in demographics or clinical risk factors between the surgical and non-operative patients (see Table 1).

**Table 1:** Surgically Treated Versus Non-Operatively Treated Patients BMI: body mass index; WBC: white blood cell; CRP: C-reactive protein; ESR: erythrocyte sedimentation rate; Hgb: hemoglobin; IV: intravenous

	Surgically Treated (n = 103)	Nonoperatively Treated (n = 44)	p-value
Mean Age (years)	57.17 ± 12.6	59.98 ± 16.2	0.310
Sex			0.947
Male (n)	72	31	
Female (n)	31	13	
Race/Ethnicity			0.199
White (n)	50	20	
Hispanic (n)	33	13	
Black (n)	8	3	
Asian (n)	7	1	
Other (n)	3	2	
Multiple (n)	2	5	
Insurance			0.563
Private (n)	23	7	
Government (n)	79	36	
Uninsured (n)	1	0	
Lives in a Medically Underserved Area (n)	25	13	0.516
Lives in a Primary Care Shortage Area (n)	40	16	0.754
Mean Household Income by Zip Code (Dollars)	$81,784.33 ± $22,359.04	$80,476.42 ± 18,006.14	0.735
Percentage of People Living in Poverty by Zip Code (%)	17.41% ± 5.59%	15.41% ± 4.10%	0.098
BMI (kg/m^2^)	29.70 ± 8.3	27.27 ± 6.4	0.092
Mean Initial WBC	12.64 ± 6.6	11.40 ± 6.5	0.303
Mean Initial CRP	11.92 ± 9.8	11.80 ± 9.2	0.945
Mean Initial ESR	88.34 ± 38.7	80.14 ± 29.0	0.262
Mean Initial Albumin	3.01 ± 0.71	3.06 ± 0.73	0.695
Mean Initial Hgb	10.80 ± 2.1	11.38 ± 2.0	0.140
Mean Length of Stay (Days)	17.4 ± 13.5	13.13 ± 9.99	0.075
History of Diabetes (n)	50	15	9.159
History of Chronic Steroid Use (n)	1	2	0.138
History of IV Drug Use (n)	26	9	0.606
Current Smoker (n)	26	12	0.953
Surgical Follow-up			< .001
≥90 Days (n)	58	9	
<90 Days (n)	45	35	
Infectious Disease Follow-up			0.993
≥90 Days (n)	21	35	
<90 Days (n)	82	9	

Surgery follow-up: ≥90 days versus <90 days

Surgery follow-up was analyzed, comparing patients who had at least 90 days of follow-up to those who had less than 90 days. Sixty-two of 147 (42.2%) patients had inadequate follow-up (<90 days) with their surgical team. Patients who had at least 90 days of follow-up with surgery were also more likely to have more than 90 days of follow-up with ID (p-value = 0.003). The only other statistically significant difference between these groups was the initial presenting hemoglobin with higher initial hemoglobin patients more likely to have adequate (≥90 days) follow-up. There were no other significant differences in demographics or clinical risk factors between the two groups (see Table 2).

**Table 2:** Surgical Follow-up, ≥90 Days Versus <90 Days BMI: body mass index; WBC: white blood cell; CRP: C-reactive protein; ESR: erythrocyte sedimentation rate; Hgb: hemoglobin; IV: intravenous

	≥90 days Follow-up with Surgery (n = 67)	<90 days Follow-up with Surgery (n = 80)	p-value
Mean Age (years)	58.45 ± 13.57	57.65 ± 14.09	0.728
Sex			0.703
Male (n)	48	55	
Female (n)	19	25	
Race/Ethnicity			0.223
White (n)	33	37	
Hispanic (n)	21	25	
Black (n)	5	6	
Asian (n)	7	1	
Other (n)	0	5	
Multiple (n)	1	6	
Insurance			0.347
Private (n)	16	14	
Government (n)	50	65	
Uninsured (n)	1	0	
Lives in a Medically Underserved Area (n)	14	24	0.213
Lives in a Primary Care Shortage Area (n)	27	29	0.595
Mean Household Income by Zip Code (Dollars)	$82,342.98 ± $20,867.79	$80,605.71 ± $21, 408.10	0.623
Percentage of People Living in Poverty by Zip Code (%)	17.08% ± 5.35%	16.75% ± 5.20%	0.721
BMI (kg/m^2^)	29.17 ± 7.85	28.82 ± 7.85	0.793
Mean Initial WBC	12.27 ± 6.01	12.29 ± 7.08	0.980
Mean Initial CRP	11.22 ± 8.61	12.46 ± 10.40	0.470
Mean Initial ESR	82.71 ± 37.12	88.69 ± 35.56	0.370
Mean Initial Albumin	3.13 ± 0.68	2.93 ± 0.73	0.104
Mean Initial Hgb	11.38 ± 2.22	10.61 ± 1.94	0.028
Mean Length of Stay (Days)	14.75 ± 8.09	17.47 ± 15.74	0.220
History of Diabetes (n)	28	37	0.496
History of Chronic Steroid Use (n)	1	2	0.643
History of IV Drug Use (n)	14	21	0.374
Current Smoker (n)	15	16	
Infectious Disease Follow-up			0.003
≥90 Days (n)	21	9	
<90 Days (n)	46	71	

Infectious disease follow-up: ≥90 days versus <90 days

ID follow-up was analyzed, comparing patients who had at least 90 days of follow-up to those who had less than 90 days. Out of 147, 112 (76.2%) patients had inadequate follow-up (<90 days) with ID. Patients who had at least 90 days of follow-up with ID were also more likely to have more than 90 days of follow-up with surgery (p-value = 0.003). Age was also significantly different between these groups as age with younger patients were more likely to have adequate (≥90 days) ID follow-up (p-value <0.001). Patients with diabetes were less likely to have adequate ID follow-up. The final two statistically significant factors were initial hemoglobin and initial albumin with patients having higher initial hemoglobin and higher albumin more likely to have ≥90 days of ID follow-up. There were no other significant differences in demographics or clinical risk factors between the two groups (see Table 3).

**Table 3:** Infectious Disease Follow-up, ≥90 days Versus <90 Days BMI: body mass index; WBC: white blood cell; CRP: C-reactive protein; ESR: erythrocyte sedimentation rate; Hgb: hemoglobin; IV: intravenous

	≥90 days Follow-up with Infectious Disease (n = 56)	<90 days Follow-up with Infectious Disease (n = 91)	p-value
Mean Age (years)	49.37 ± 16.31	60.25 ± 12.21	< .001
Sex			0.367
Male (n)	19	84	
Female (n)	11	33	
Race/Ethnicity			0.121
White (n)	12	58	
Hispanic (n)	15	31	
Black (n)	1	10	
Asian (n)	2	6	
Other (n)	0	5	
Multiple (n)	7	7	
Insurance			0.580
Private (n)	8	22	
Government (n)	22	93	
Uninsured (n)	0	1	
Lives in a Medically Underserved Area (n)	6	32	0.492
Lives in a Primary Care Shortage Area (n)	13	43	0.380
Mean Household Income by Zip Code (Dollars)	$81,590.90 ± $22, 178.57	$81,345.75 ± $20,920.78	0.955
Percentage of People Living in Poverty by Zip Code (%)	17.34% ± 5.61%	16.79% ± 5.15%	0.544
BMI (kg/m^2^)	29.50 ± 8.98	28.85 ± 7.53	0.692
Mean Initial WBC	12.73 ± 5.75	12.16 ± 6.80	0.673
Mean Initial CRP	12.03 ± 10.02	11.85 ± 9.52	0.930
Mean Initial ESR	95.96 ± 30.16	82.90 ± 37.53	0.095
Mean Initial Albumin	3.35 ± 0.81	2.94 ± 0.67	0.005
Mean Initial Hgb	11.76 ± 2.07	10.76 ± 2.07	0.021
Mean Length of Stay (Days)	15.6 ± 9.06	16.31 ± 13.53	0.796
History of Diabetes	8	57	0.025
History of Chronic Steroid Use	1	2	0.590
History of IV Drug Use	8	27	0.735
Current Smoker (n)	8	30	0.877
Surgical Follow-up			0.003
≥90 Days (n)	21	46	
<90 Days (n)	9	71	

Expected follow-up: surgically treated versus non-operatively treated

Finally, an analysis of surgically treated versus non-operatively treated patients and their 'expected' follow-up rates were performed. Surgically treated patients were significantly more likely to achieve ≥90 days of expected follow-up with Orthopedic Surgery/Neurological Surgery than non-operatively treated patients with ID (p-value < 0.001) (see Table 4).

**Table 4:** Expected Follow-up for Surgically Versus Non-Operatively Treated Patients

	Surgically Treated (n = 103)	Non-Operatively Treated (n = 44)	p-value
≥90 days Follow-up with Expected Service (n)	58	9	< .001
<90 days Follow-up with Expected Service (n)	45	35	

## Discussion

Upon review of much of the SEA literature, the authors of this study noted that there was not a lot of available detail regarding patient follow-up in a majority of the publications. Most often, follow-up was reported as a mean and a range, with further details omitted. It was also typically not clearly reported how many patients may have been excluded from the study due to inadequate follow-up [2,4,5,9-19]. Retrospective studies using the National Surgical Quality Improvement Program (NSQIP) database only include follow-up up to 30 days [20,21].

From the data we do have, there does appear to be a varying degree of difficulty with patient follow-up in the SEA literature. Karkari et al. reported an average of 39.6 weeks of follow-up (with four patients lost to follow-up) but a very broad range of one to 444 weeks, also noting short follow-up periods of less than six weeks in a portion of their patients as one of the limitations of their study [22]. Adogwa et al. had a similarly broad range of follow-up with a mean of 41.38 ± 86.48 weeks and four of 82 patients lost to follow-up [23]. Alton et al. showed an average follow-up of 233.9 days with an SD of 402.7, a range of three to 1797 days [24]. Keller et al. noted that only 36 of 154 total patients had a six months follow-up evaluation available for their review [25]. Eighty-nine of 339 patients (26.3%) in the Shah et al. 2019 paper had follow-ups of one year or more [26]. Baum et al. reported a loss to follow-up rates (< one year of follow-up) of 15.9% and 21.4% in non-intravenous drug users versus intravenous drug users respectively [27]. Other studies reported seemingly better success with their follow-up. The mean follow-up for Khanna et al. was 20.9 months (with a range of 4-45 months) [28]. Lenga et al. reported a mean follow-up of 26.6 ± 12.4 months [12].

This retrospective study demonstrated shockingly poor follow-up for all SEA patients at a single institution. Patients with higher initial hemoglobin and albumin had better follow-ups. These values could potentially be a marker for overall health/nutrition and socioeconomic status which could directly correlate to the patient’s overall propensity and/or ability to reliably follow-up. It is more difficult to elucidate implications related to the significance of age and diabetes status noted in the ID follow-up group.

The primary statistically significant factor affecting follow-up in the current study was surgical status, with surgically treated patients having better follow-up rates than those treated non-operatively. This finding is important when considering treatment options, particularly in the context that non-operative patients likely require even more diligent follow-up care to monitor for potential treatment failure. Behmanesh et al. showed a 30% readmission rate in non-operatively treated patients (with many of those subsequently going on to surgical intervention) compared to a 4.1% and 6.4% readmission rate in early and late surgically treated patients respectively [14]. Pitaro et al. also noted lower readmission rates in surgically treated patients [9]. In another study, 52% of conservatively treated patients were noted to have worsened compared to 8% in the surgically treated group [15].

While many appropriately selected patients can be treated non-operatively, the literature supports aggressively treating epidural abscesses surgically when there is a corresponding clinical concern [9,14,15]. Perhaps, reliable follow-up should also be considered when formulating a treatment plan for patients with SEAs. Regimented outpatient plans may also be required to assist with follow-up care. This could include pre-scheduled follow-up and coordinated follow-up dates and times with ID specialists. A periodic dedicated 'epidural abscess clinic' with combined ID and orthopedic/neurosurgical colleagues might also be an option at tertiary care centers where the volume is feasible.

Limitations

Limitations of the study include limited patient numbers and subsequent lower power. This was a single-center study, so the results may not be generalizable. A larger multi-center study would better clarify how applicable these conclusions are broad. The institution in this study does have some notable and unique challenges. It is the lone tertiary care center in the largest county by area in the United States (20,105 square miles) with a county population of 2,194,710 [29]. Given this large area, many patients are forced to travel up to two to three hours for their care [29]. A total of 14.3% of residents live in poverty, higher than the state average [29]. Minority groups make up a majority of the county’s population with 55.8% identifying as Hispanic [29].

## Conclusions

Given the significant risk for morbidity and mortality, follow-up is critically important in the SEA patient population. Unfortunately, this study and prior literature show that follow-up rates are poor for these patients. Patients who are treated surgically may be more likely to have at least 90 days of follow-up. Reliable follow-up may be another important variable to consider when formulating a treatment plan for patients with SEAs. Extra efforts may also need to be taken at the institutional level to coordinate outpatient follow-up care.
